# Gluten encephalopathy with psychiatric onset: case report

**DOI:** 10.1186/1745-0179-5-16

**Published:** 2009-06-26

**Authors:** Nicola Poloni, Simone Vender, Emilio Bolla, Paola Bortolaso, Chiara Costantini, Camilla Callegari

**Affiliations:** 1 Department of Clinical Medicine-Psychiatry, University of Insubria, Via O. Rossi 9, 21100 Varese, Italy

## Abstract

Many cases of coeliac disease, a gastrointestinal autoimmune disorder caused by sensitivity to gluten, can remain in a subclinical stage or undiagnosed. In a significant proportion of cases (10–15%) gluten intolerance can be associated with central or peripheral nervous system and psychiatric disorders.

A 38-year-old man was admitted as to our department an inpatient for worsening anxiety symptoms and behavioural alterations. After the addition of second generation antipsychotic to the therapeutic regimen, the patient presented neuromotor impairment with high fever, sopor, leukocytosis, raised rhabdomyolysis-related indicators. Neuroleptic malignant syndrome was strongly suspected. After worsening of his neuropsychiatric conditions, with the onset of a frontal cognitive deficit, bradykinesia and difficulty walking, dysphagia, anorexia and hypoferraemic anaemia, SPET revealed a reduction of cerebral perfusion and ENeG results were compatible with a mainly motor polyneuropathy. Extensive laboratory investigations gave positive results for anti-gliadin antibodies, and an appropriate diet led to a progressive remission of the encephalopathy.

## Introduction

Coeliac disease is an inflammatory disease of the upper small intestine resulting from gluten ingestion [1]. The diagnosis is based on: a clinical picture suggesting malabsorption of nutrients, serology for anti-gliadin, anti-endomysial and anti-transglutaminase antibodies, sometimes a biopsy of the intestinal mucosa, and resolution of the lesions following the institution of a gluten-free diet [1]. Many cases of coeliac disease long remain in a subclinical stage [2], or undiagnosed because of poor awareness of the condition among primary care physicians [1]. In a significant proportion of cases (10–15%) gluten intolerance can be associated with central or peripheral nervous system disorders, such as cerebellar ataxia, myoclonus, epilepsy, ophthalmoplegia, dementia, multifocal leukoencephalopathy, peripheral neuropathies and myopathies [3] and with psychiatric disorders such as anxiety, depression, psychotic symptoms and personality disorders [4]. These manifestations are sometimes the presenting symptoms of the disease [4-6]. The physiopathological mechanisms underlying these associations are still not known, even though genetic causes [6] and autoimmune factors [7,8] have been hypothesised.

The literature describes cases of cerebral perfusion abnormalities in untreated coeliac patients [9,10]. There is also a report of a case of regression of frontal hypoperfusion following the institution of a gluten-free diet [9].

## Case report

In May 2001, a 38-year-old man with anxious-depressive symptoms was referred to us for psychiatric assessment. These symptoms, occurring sporadically for around two years, had worsened following a protracted absence from work (due to a disabling right wrist fracture). The patient had a history of surgical operations to correct kyphoscoliosis.

He was diagnosed with reactive depressive disorder in the context of personality disorder NOS (not otherwise specified) and put on paroxetine 10 mg with benzodiazepines. Following the appearance of bizarre behaviours and heteroaggressiveness towards family members, anti-psychotic therapy (haloperidol decanoate 50 mg every four weeks) was added. In May 2002, worsening anxiety symptoms and behavioural alterations that could not be managed at home culminated in the patient's hospitalisation in our department for re-assessment and review of therapy. Two days after the addition of risperidone 2 mg to the existing therapeutic regimen (citalopram 20 mg and BDZ) the patient presented muscle rigidity, cramp-like muscle pain and increased osteo-tendinous reflexes leading to bradykinesia and difficulty walking. Withdrawal of the anti-psychotic drug did not improve the picture significantly. Laboratory investigations revealed raised CK (536 U/l) and a brain CT-scan showed an area of hypodensity of possible ischaemic origin in the posterior fossa, as well as moderate deepening of the cortical sulci in the frontal-temporal region bilaterally. EEG showed mild, non-specific, non-focal abnormalities.

The severe anxiety symptoms and behavioural alterations persisted and a week later anti-psychotic treatment was re-introduced. The clinical picture, already characterised by neuromotor impairment, worsened abruptly and unexpectedly, with the onset of high fever (39°C), sopor, acute respiratory insufficiency with peripheral cyanosis, leukocytosis (WBC count 16680/mm^3^), and raised rhabdomyolysis-related indicators (CK 1216 U/l and LDH 718 U/l). The patient was transferred to the infectious diseases department. Since neuroleptic malignant syndrome was strongly suspected, the anti-psychotic was withdrawn and dantrolene 50 mg/day and cardiorespiratory support were started, substantially resolving the acute symptomatology. The patient developed an *Enterococcus faecalis *infection of the urinary tract and deep-vein thrombosis (DVT), which were treated with antibiotic therapy and subcutaneous heparin.

Although the patient's general conditions improved, the neurological picture of diffuse muscle rigidity, psychomotor slowing and dysarthria persisted and despite further investigations (MRI, evaluation of autoantibodies and circulating immunocomplexes) continued to lack a plausible explanation.

In mid-June, a further worsening of his neuropsychiatric conditions prompted his readmission to our department. During this second stay, he displayed the progressive onset of a frontal cognitive deficit together with affective lability, behavioural and affective regression, and verbal and motor stereotypes. Furthermore, the appearance of dysphagia and anorexia led to significant weight loss (20 kg in two months) which necessitated parenteral nutrition.

The patient was then sent to the Neuropathology Unit at the "C. Besta" Neurological Institute in Milan, where single photon emission computed tomography (SPECT) revealed a reduction of perfusion and thus of neuronal activity and density in the right superior and middle frontal gyri, the left superior frontal gyrus, and the left medial temporal and occipital gyri; electroneurography (ENeG) results were compatible with a mainly motor polyneuropathy. Subsequently, mild hypoferraemic anaemia was found. Gluten sensitivity tests (part of further and more extensive laboratory investigations) gave positive results for anti-gliadin (IgG 32 UI/ml), anti-endomysial and anti-transglutaminase antibodies). A appropriate diet was instituted and led to a progressive remission of the encephalopathy and an improvement in the psychiatric symptoms and the lesions detected on SPECT and ENeG, which, at follow up, were no longer present. After a period of rehabilitation, this patient is still followed by our psychiatric service for mild anxiety symptoms. He takes olanzapine 2.5 mg and derives benefit from the treatment.

## Conclusion

The diagnostic process in this patient proved particularly complicated. This is, in fact, a case of clinical onset of coeliac disease in adulthood, without signs of malabsorption and with exclusively psychiatric involvement (non-specific anxious-depressive symptoms associated with affective and behavioural personality disorders). In our view, this clinical picture could be attributed to the SPET-documented frontal hypoperfusion. This would indeed explain the lack of benefit of the initial psychopharmacological treatment and the progressive worsening of the symptoms that, together with the onset of behavioural disinhibition, necessitated the patient's hospitalisation. During hospitalisation, the administration of anti-psychotic drugs triggered the onset of neuroleptic malignant syndrome, which, initially atypical (without fever and leukocytosis) and then full blown, slowed down the diagnostic process and delayed the recognition of the true nature (organic) of the aetiology. We cannot rule out the possibility that this coeliac patient presented a particular susceptibility to this rare complication associated with the use of second-generation antipsychotic drugs [11], given the cerebral involvement documented on brain CT-scan and subsequently on SPECT. The picture was complicated further by the onset of DVT and a urinary tract infection, probably due to the patient's prolonged confinement to bed.

After the resolution of the acute picture, there remained a progressively worsening psycho-organic syndrome, secondary to the above-mentioned cerebral involvement, and neuromotor deficits due to the polyneuropathy detected on ENeG. The case we describe recalls literature reports of an adult-onset progressive frontal, subcortical-type cognitive deficit, characterised by confusion, personality disturbances and associated neurological (ataxia- and peripheral neuropathy-type) pictures, coinciding with the exacerbation of a malabsorption syndrome [12].

The appearance of the first signs pointing to malabsorption, i.e., the weight loss and hypoferraemic anaemia, finally prompted us to investigate a possible autoimmune aetiology, and to test for anti-gliadin antibodies, for which the patient was positive.

The diagnostic hypothesis of gluten encephalopathy was confirmed by the remission of the symptoms and of the lesions observed on SPECT following the institution of an appropriate diet.

In addition to the objective diagnostic difficulties presented by this case, we wish to add a further, ideological consideration, relating to an unwitting tendency of colleagues from other specialist disciplines to stigmatise patients classified as "psychiatric". Indeed, both the possible organic aetiology of the clinical picture and the concomitant medical disorders presented by "psychiatric" patients are often underestimated, slowing down the diagnostic process and the taking of the necessary therapeutic measures.

## Consent

Written informed consent was obtained from the patient for publication of this case report and any accompanying images. A copy of the written consent is available for review by the Editor-in-Chief of this journal.

## Competing interests

The authors declare that they have no competing interests.

## Authors' contributions

NP has made substantial contributions to the acquisition of clinical data; VS has given the final approval of the version to be published; BE has made substantial contributions to the acquisition of clinical data; PB participated in the analysis and interpretation of clinical data; CC was involved in drafting the manuscript; CC was involved in revising the manuscript critically.

All authors read and approved the final manuscript.
